# Novel Angiogenic Regulators and Anti-Angiogenesis Drugs Targeting Angiogenesis Signaling Pathways: Perspectives for Targeting Angiogenesis in Lung Cancer

**DOI:** 10.3389/fonc.2022.842960

**Published:** 2022-03-16

**Authors:** Yingying Li, Mengmeng Lin, Shiyuan Wang, Bo Cao, Chunyu Li, Guohui Li

**Affiliations:** Pharmacy Department, National Cancer Center/National Clinical Research Center for Cancer/Cancer Hospital, Chinese Academy of Medical Sciences and Peking Union Medical College, Beijing, China

**Keywords:** lung cancer, angiogenesis, angiogenesis signaling pathway, angiogenic regulator, anti-angiogenesis drug

## Abstract

Lung cancer growth is dependent on angiogenesis. In recent years, angiogenesis inhibitors have attracted more and more attention as potential lung cancer treatments. Current anti-angiogenic drugs targeting VEGF or receptor tyrosine kinases mainly inhibit tumor growth by reducing angiogenesis and blocking the energy supply of lung cancer cells. However, these drugs have limited efficiency, raising concerns about limited scope of action and mechanisms of patient resistance to existing drugs. Therefore, current basic research on angiogenic regulators has focused more on screening carcinogenic/anticancer genes, miRNAs, lncRNAs, proteins and other biomolecules capable of regulating the expression of specific targets in angiogenesis signaling pathways. In addition, new uses for existing drugs and new drug delivery systems have received increasing attention. In our article, we analyze the application status and research hotspots of angiogenesis inhibitors in lung cancer treatment as a reference for subsequent mechanistic research and drug development.

**Graphic Abstract:**
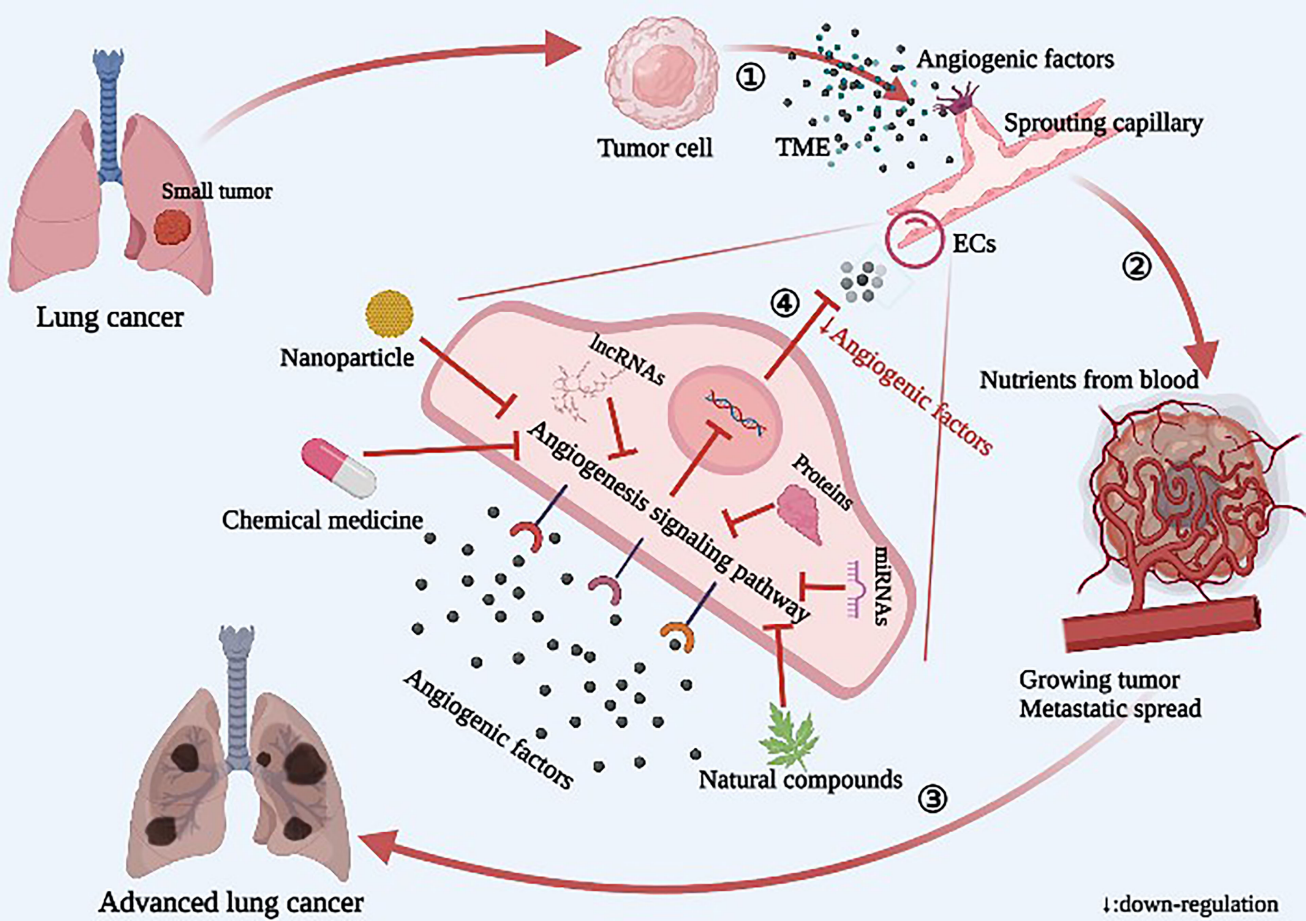
Diagram of the relationship between angiogenesis and tumor growth and metastasis in lung cancer. ① The tumor releases a large number of angiogenic factors into the tumor microenvironment (TME), resulting in sprouting of endothelial cells (ECs) and increased angiogenesis. ② Neovascularization provides nutrients for tumor growth, and the tumor rapidly grows and spreads. ③ In lung cancer, the local tumor spreads to the whole lung, including the blood, and then spreads to the whole body. ④ Novel angiogenesis inhibitors inhibit angiogenesis by acting on specific targets of signaling pathways.

## Introduction

Lung cancer is a malignant tumor with high morbidity and mortality worldwide. The incidence of lung cancer in males is significantly higher than that in females. Smoking is the main reason for the high incidence of lung cancer. Clinically, lung cancer can be generally divided into non-small cell lung cancer (NSCLC) and small cell lung cancer (SCLC), of which NSCLC accounts for up to 80-85% (1, 2). There are significant differences between these two subtypes in histological type, biological behavior, morbidity, prognosis and response to treatment. Since lung cancer is often diagnosed in the advanced stage, patients with lung cancer cannot receive timely and effective treatment; therefore, the overall survival rate is very low (3, 4). As a result, it is necessary to find new treatment methods to treat lung cancer. Studies have shown that the dynamic balance between pro-angiogenic factors and anti-angiogenic factors in the tumor microenvironment (TME) is often broken in cancer. Tumor cells secrete a large amount of pro-angiogenic factors, leading to a pro-angiogenic orientation, which removes wastes and toxic substances while meeting tumor metabolic requirements. Therefore, controlling angiogenesis may be an effective means for treatment of malignant tumors (5–7). Lung cancer growth depends on angiogenesis, and massive angiogenesis is associated with lung cancer invasion and poor prognosis. In recent years, anti-angiogenesis drugs have exhibited good clinical efficacy for lung cancer patients, leading anti-angiogenic strategies to be widely studied with respect to lung cancer treatment (8).

VEGF, the first discovered angiogenic factor, regulates almost all angiogenesis signaling pathways, and current anti-angiogenesis drugs primarily include monoclonal antibodies targeting VEGF and tyrosine kinase inhibitors (TKIs) clinically developed for VEGFR and EGFR. Monoclonal antibodies mainly include Bevacizumab and Ramuzumab, which target VEGF-A and prevent it from binding with the VEGFR to exert its anti-angiogenic role (9). Among TKIs developed for VEGFR, Apatinib and Pazopanib act on the single target of VEGFR-2, while Regorafenib and Sorafenib act on both VEGFR-2 and VEGFR-3. Sunitinib acts on VEGFR-1, VEGFR-2 and VEGFR-3, leading to a stronger inhibitory effect on VEGF/VEGFR angiogenesis signaling. EGFR is overexpressed in 50% of NSCLC patients and can activate Ras/Raf/MEK/ERK and PI3K/AKT/mTOR signaling pathways, with EGFR mutations very common in advanced NSCLC (10). The first generation of EGFR-TKIs developed for EGFR includes Gefitinib and Icotinib, which can only inhibit a single target (EGFR) with limited ability. Moreover, EGFR mutations (such as the most common T790M mutation) will occur after extended use of these drugs, resulting in significant drug resistance. Therefore, the second and third generations of EGFR-TKIs emerged at the right moment. The second generation of EGFR-TKIs contains Afatinib and Dacomitinib, which can synergistically inhibit EGFR, HER2 and HER4, while also inhibiting tumor cells with the most common exon 19 deletion mutation and exon 21 (L858R) mutation in the EGFR gene (11). The third generation of EGFR-TKIs contains Osimertinib, which is a typical multi-target TKI with stronger inhibitory effects on angiogenesis that can deal well with the occurrence of various mutations. Osimertinib has inhibitory effects on mutant EGFR (including exon 19 and 21 deletions and T790M), as well as on HER2, HER3 and HER4. However, currently anti-angiogenesis drugs have significant limitations preventing clinical application. First, these drugs only inhibit nutritional TME, rather than directly killing the tumor itself, so their function is limited. Second, drug resistance is a key issue for current treatment strategies that inhibit vascular production, including increased or mutated expression of target molecules and activation of abnormal or alternative signaling pathways. Once a tumor is resistant, it may actively reshape its growth environment, thus rendering drugs acting on vascular endothelial cells (ECs) ineffective (12). Third, the toxicity and side effects of anti-angiogenesis drugs should not be underestimated. Toxicity to skin, digestive tract, liver, kidney and other systems should be given special attention, as angiogenic factors also promote immunosuppression. These immunosuppressive cells can stimulate angiogenesis in turn, creating a vicious cycle conducive to tumor growth (13). Therefore, a combination of reasonable amounts of anti-angiogenesis drugs and immune checkpoint inhibitors (ICIs) not only can inhibit the blood vessel production vital to tumor progression, but also, by normalizing tumor blood vessels to relieve tumor hypoxia, simultaneously lower the level of immune checkpoints to block immune inhibitory signals, reducing the occurrence of tumor immune escape. In addition, the application of ICIs can also improve the efficacy of anti-angiogenesis therapy by recruiting immune cell subtypes with vascular regulation functions (14). The combination of anti-angiogenesis drugs and ICIs will greatly increase the efficacy of anti-tumor therapy. Therefore, anti-angiogenesis drugs are often combined with conventional chemotherapy, radiotherapy or immunotherapy in clinical practice to achieve better efficacy. In the long term, combined therapy can also reduce the occurrence of drug resistance. In view of the status of angiogenesis inhibitors in lung cancer treatment, current basic research regarding angiogenic regulators focuses primarily on screening carcinogenic/anticancer genes, miRNAs, lncRNAs, proteins and other biomolecules to regulate the expression of specific targets in the angiogenesis signaling pathway, ultimately attempting to inhibit activation of the angiogenesis signaling pathway at the root. This deepening understanding of angiogenic signaling pathways provides a theoretical basis for rational drug design. In addition, current anti-angiogenesis drugs have limited efficacy and high cost; therefore, the repurposing of existing drugs is important to not only achieve efficacy but also save money. Furthermore, new preparation technology can significantly improve drug bioavailability and reduce drug toxicity and side effects, drawing increased attention to the research and development of new dosage forms of angiogenesis inhibitors.

## Novel Angiogenic Regulators in Lung Cancer Treatment

### Angiogenesis Regulatory Axis

Currently, the signaling pathways known to be involved in lung cancer angiogenesis include VEGF/VEGFR (15, 16), HIF-1α (17), PI3K/AKT/mTOR (18), Ras/Raf/MEK/ERK (18), JAK/STAT (19), NF-κB (20), Shh (21) and DLL4/Notch (22), among others. The discovery of the angiogenesis regulatory axis in lung cancer has been largely based on the elucidated signaling pathways. Through the study of some key targets in the pathway, activators or inhibitors of this target are found, and a new angiogenesis regulatory axis is derived.

**Figure 1:**
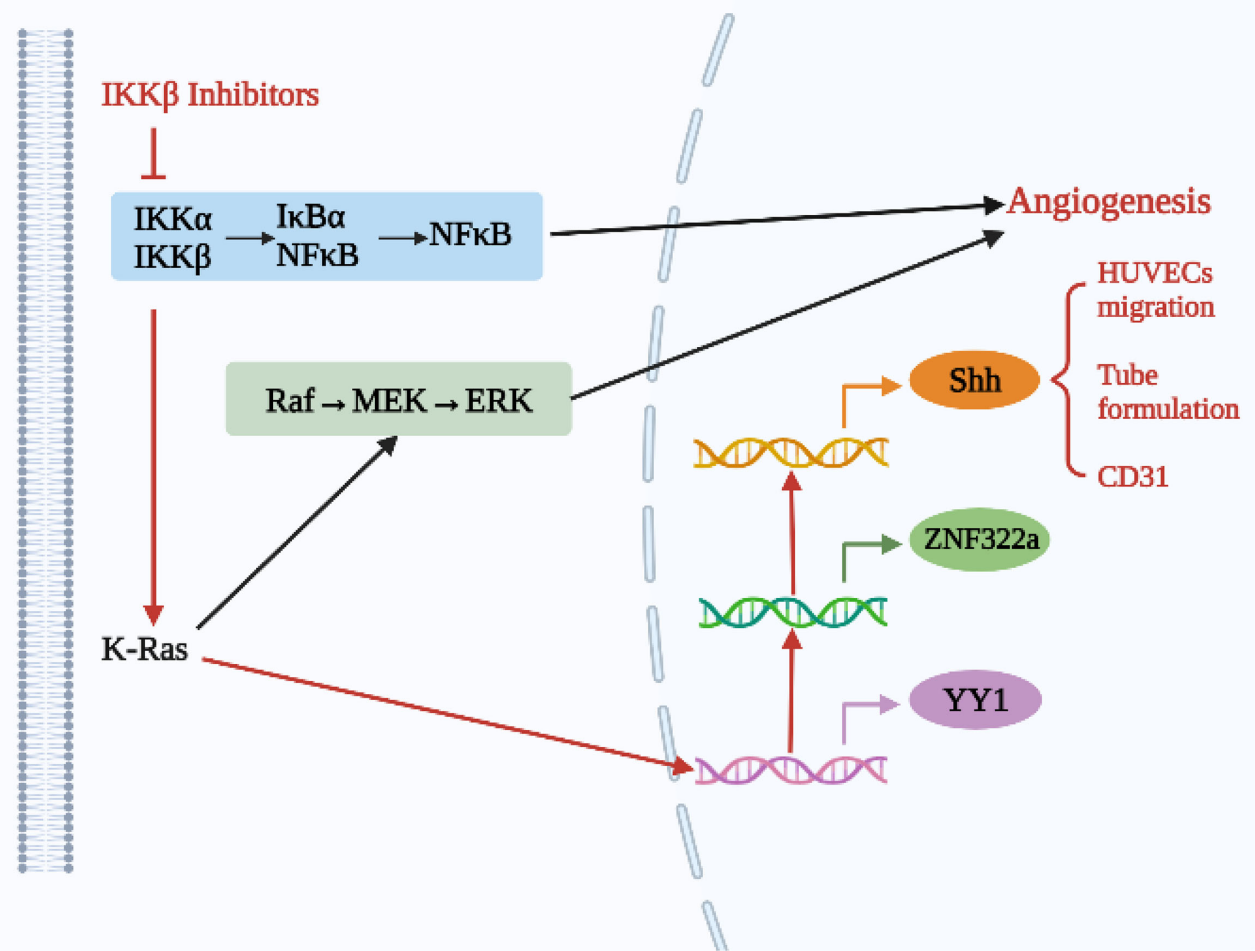
Novel regulatory axis for inhibiting angiogenesis in lung cancer.

As shown in **Figure 1**, the K-Ras/YY1/ZNF322A/Shh regulatory axis may be an important mechanism of neovascularization (23). Increased expression of carcinogenic K-Ras enhances the expression of Yin Yang 1 (YY1), a transcription factor that directly activates transcription of zinc finger protein 322A (ZNF322A). Subsequently, overexpressed ZNF322A binds to the Shh promoter to enhance Shh expression. Activation of the transcription axis can promote EC migration, tube formation and CD31 expression *in vitro*, suggesting that K-Ras/YY1/ZNF322A regulatory axis-mediated Shh activation can promote *in vitro* and *in vivo* angiogenesis. Therefore, therapeutic strategies targeting the K-Ras/YY1/ZNF322A/Shh regulatory axis may provide new ideas for targeted treatment of lung cancer patients. CD31 is an endothelial marker that plays important roles in the angiogenesis and metastasis of lung cancer. IκB kinase (IKK) phosphorylates serine at the IκB subtone site of the intracellular NF-κB·IκB complex, resulting in ubiquitination of the IκB subunit, subsequent degradation by proteases, and release of the NF-κB dimer. The free NF-κB dimer enters the nucleus and promotes transcription of related genes, such as c-Myc, matrix metalloproteinase-9 (MMP-9), VEGF, and others (20). Matrix metalloproteinases-2 and 9 (MMP-2 and MMP-9) are closely related to EC function and angiogenesis in lung cancer, so both are often used as important indicators of angiogenesis. Carnero-lobo et al. (24) found that IKKβ promotes K-Ras-induced angiogenesis through both inherent and cancer-independent mechanisms, suggesting that IKKβ inhibition is a promising anti-angiogenesis approach. IKKβ inhibition can reduce the expression of VEGF and IL-8 downstream of different K-Ras signaling pathways, further inhibiting ECs activation and angiogenesis and confirming potential use for the treatment of K-Ras-induced lung cancer.

### Genes

Astrocyte elevating gene-1 (*AEG-1*) is involved in the tumorigenesis and angiogenesis of various human malignant diseases. However, the role of *AEG-1* in the angiogenesis of NSCLC remains to be further clarified. Ma et al. (25) showed that up-regulation of *AEG-1* mRNA in NSCLC is associated with increased tumor angiogenesis. *In vitro*, studies have shown that *AEG-1* promotes tube formation, increases invasion of human umbilical vein endothelial cells (HUVECs), enhances *AEG-1* expression in tumor slices, and further enhances the expression of specific angiogenic molecules, including VEGF, Ang1, MMP-2 and HIF-1α, through the PI3K/AKT signaling pathway. Therefore, *AEG-1* is a gene that potentially promotes angiogenesis in lung cancer by regulating the PI3K/AKT signaling pathway.

### MiRNAs

In recent years, the role of microRNA (miRNA or miR) in lung cancer treatment has been extensively studied (26, 27). miRNAs are a group of highly conserved small non-coding RNAs of 21-25 nucleotides in length. By binding to complementary sequences in the 3’UTRs of target messenger RNAs, miRNAs regulate gene expression post-transcriptionally, ultimately leading to translation inhibition and reduced protein expression (28). New evidence suggests that a variety of human cancers, including lung cancer, exhibit abnormal miRNA expression. MiRNAs can function as both carcinogens and tumor suppressors. In NSCLC, miRNAs are involved in cell proliferation, migration and tumor angiogenesis (29, 30). Through overexpression of suppressive miRNAs or inhibition of oncogenic miRNAs, it is possible to completely block all specific target-associated angiogenic signaling pathways; therefore, miRNAs have been widely studied in the field of lung cancer treatment.

**Figure 2:**
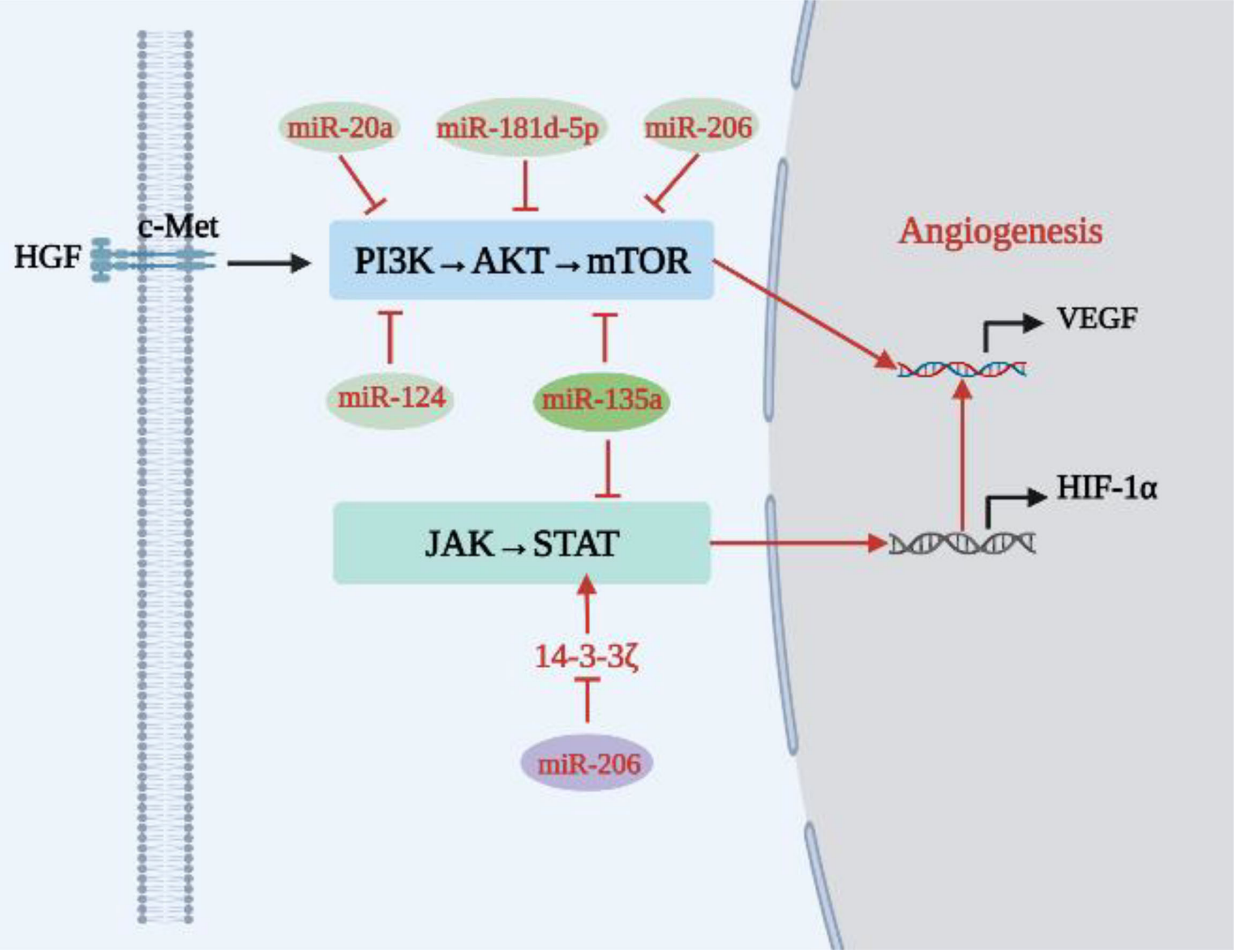
Relationship between tumor suppressor miRNAs and angiogenesis in lung cancer.

As shown in **Table 1** and **Figure 2**, some miRNAs function as cancer suppressors *in vivo*.

**Table 1:** Research progress of miRNAs in angiogenesis in lung cancer.

↑miRNAs	Signaling pathway	Angiogenesis indication
miR-20a (31) miR-181d-5p (32)	↓PI3K/AKT	↓angiogenesis
miR-206 (33)	↓HGF/c-Met ↓c-Met/PI3K/AKT/mTOR	↓c-Met, AKT, mTOR ↓HUVECs, VEGF
miR-135a (34, 35)	↓IGF-1/PI3K/AKT ↓STAT6	↓VEGF, bFGF, IL-8 ↓COX-2, c-Myc, JAK2, ROCK1
miR-206 (36)	↓14-3-3ζ/STAT3/HIF-1α/VEGF	↓VEGF, Ang2, IL-8
miR-124 (37)	↓AKT	↓cell proliferation
miR-210 (38, 39)	↑RUNX3/PI3K/AKT ↑JAK/STAT3	↑HUVECs ↑angiogenesis
miR-301a (19)	↑JAK/STAT3	↑VEGF, MMP-9
miR-494 (40)	↑HIF-1α/miR-494/AKT/eNOS	↑angiogenesis.

↑: up-regulation ↓: down-regulation.

Compared with adjacent tissues, expression of ribonucleotide reductase regulatory subunit M2 (RRM2) was up-regulated in cancer tissues of NSCLC patients, while expression of miR-20a was down-regulated. miR-20a directly targets the A549 cell line and the RRM2-mediated PI3K/AKT signaling pathway in HUVECs to inhibit NSCLC cell proliferation, EC migration, and tube formation (31). In addition, miR-181d-5p can negatively regulate the PI3K/AKT signaling pathway and inhibit angiogenesis in lung cancer (32). MiR-206 is down-regulated in different types of cancer, including lung cancer (41, 42). Chen et al. (33) found that miR-206 inhibits proliferation of HUVECs and reduces expression of Met, AKT, mTOR and VEGF in HGF-stimulated HUVECs through the c-Met/PI3K/AKT/mTOR signaling pathway, therefore inhibiting NSCLC angiogenesis. Similarly, miR-135a can block the IGF-1/PI3K/AKT signaling pathway in A549 cells, inhibit the migration and invasion of A549 cells, reduce expression of VEGF, bFGF and IL-8 in A549 cells, and inhibit angiogenesis in lung cancer (34). Shi et al. (35) reached a similar conclusion, finding that miR-135a inhibited migration and invasion of NSCLC cells by acting on KLF-8 and that miR-135a played a tumor-suppressive role by negatively regulating c-Myc, JAK2 and ROCK1 (43–45). C-Myc is an oncogene that can enhance tumor invasiveness and is a cause of poor prognosis in NSCLC patients. Activation of JAK2 promotes tumor angiogenesis *in vitro*, and inhibition of ROCK1 inhibits the growth and invasion of NSCLC cells. In addition, miR-135a has the ability to directly target STAT6 to induce apoptosis (46), and STAT6 can promote tumor development by increasing tumor invasion, inhibiting apoptosis, and promoting tumor angiogenesis-mediated COX-2 expression (47). In conclusion, miR-135a is an effective anti-lung cancer angiogenic RNA that can negatively regulate the PI3K/AKT and JAK/STAT signaling pathways and reduce transcription and expression levels of angiogenic factors. In addition, Xue et al. (36) found that 14-3-3ζ can enhance phosphorylation of STAT3 and promote secretion of VEGF, ANG-2 and IL-8. However, miR-206 overexpression reduces the expression of 14-3-3ζ, STAT3, HIF-1α and VEGF by blocking the 14-3-3ζ/STAT3/HIF-1α/VEGF signaling pathway, weakening angiogenesis and tumor growth of NSCLC *in vivo*. Therefore, up-regulating the expression of miR-206 and down-regulating the expression of 14-3-3ζ can strongly block the 14-3-3ζ/STAT3/HIF-1α/VEGF signaling pathway and inhibit angiogenesis. Similarly, miR-124 reduced AKT1/2 expression by inhibiting AKT targets in the PI3K/AKT signaling pathway associated with angiogenesis (37).

**Figure 3:**
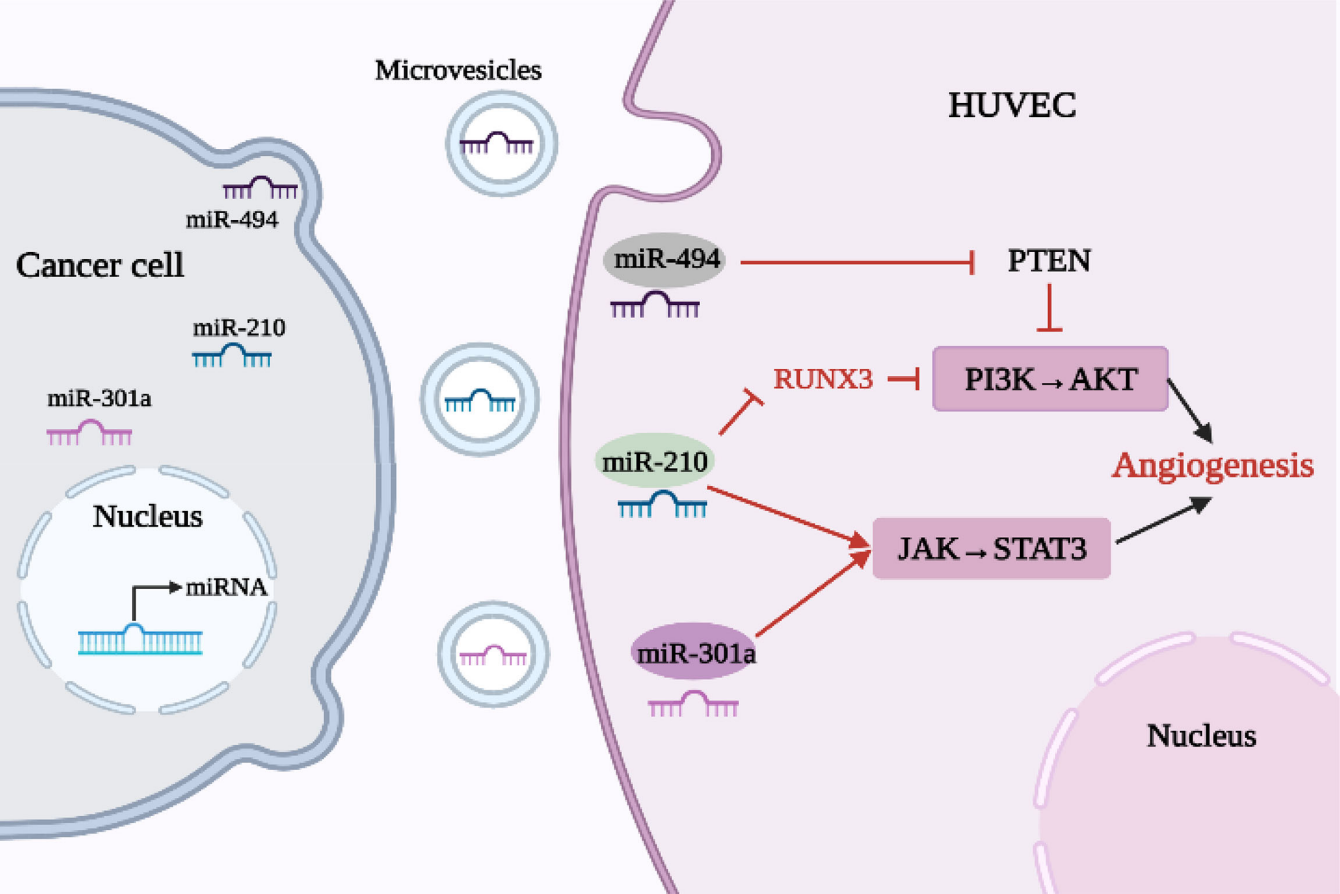
Relationship between oncogenic miRNAs and angiogenesis in lung cancer.

Conversely, as shown in **Table 1** and **Figure 3**, some highly expressed miRNAs act as angiogenic factors *in vivo*.

Exosomes are involved in a number of processes related to lung cancer, such as cell proliferation, metastasis and angiogenesis. Tumor-derived exosomes are involved in the formation and progression of lung cancer through the delivery of functional biomolecules, including miRNAs. Lung cancer cell-derived exosome miR-210 is highly expressed in NSCLC, promoting HUVEC proliferation and angiogenesis. However, miR-210 inhibitors antagonized the biological characteristics of A549 and H460 cells and reduced cell proliferation, migration and invasion. Li et al. (38) found that increased expression of the transcription factor RUNX3 can weaken the PI3K/AKT signaling pathway. Exosome miR-210 can target and negatively regulate expression of RUNX3, promoting lung cancer angiogenesis and tumor development by enhancing the PI3K/AKT signaling pathway. In addition, miR-210 may also inhibit tumorigenesis by regulating the JAK2/STAT3 pathway (39). Therefore, if miR-210 levels can be down-regulated, angiogenesis will be attenuated. It has also been shown that miR-301a increased downstream expression of VEGF and MMP-9 by activating the JAK/STAT3 signaling pathway and promoted tumor angiogenesis, invasion, and metastasis in pancreatic cancer (19). MiR-494 is an important tumor-derived miRNA that promotes tumor angiogenesis, enhancing the ability of ECs to form tubular structures on a matrix and exhibiting pro-angiogenic activity. Studies have shown that, due to the stimulation of HIF-1α, which is highly expressed in lung cancer, miR-494 is upregulated and secreted by tumor cells into the TME before entering ECs through vesicles, where it promotes tumor development by down-regulating PTEN (an inhibitor of AKT) and activating the AKT/eNOS pathway (40). In addition, some other carcinogenic miRNAs have been found. For example, miR-93-5p plays a carcinogenic role in NSCLC (48), upregulation of miR-1204 promotes cell proliferation by targeting Paired-Like Homeodomain 1 (PITX1) in NSCLC (49), miR-543 promotes NSCLC development and angiogenesis by regulating metastasis associated protein 1 (MTA1) (50), and overexpression of miR-141 in NSCLC can promote the formation of HUVEC tubes and increase angiogenesis (51).

With regard to tumor suppressor miRNAs, their expression is generally low in lung cancer tissues. If expression of tumor suppressor miRNAs can be upregulated, the angiogenesis pathways regulated by miRNAs can be further inhibited. Oncogenic miRNAs, which are highly expressed in tumor tissues, are tumor-derived and delivered in vesicles secreted by tumor cells, so their expression should be attenuated. Compared with TKIs, miRNAs are advantageous because they regulate signal pathways at the root, have stronger effects with less toxicity and fewer side effects, and act more rapidly. In addition, compensatory pathways play weaker roles, and new drug delivery systems can be developed to promote development. However, research regarding miRNAs remains at the molecular and cellular level; only a small number of miRNAs have been tested on animals, and this research is still immature. Ultimately, techniques to safely, accurately and effectively increase or inhibit levels of relevant miRNAs represent the key to treatment.

### LncRNAs

Long non-coding RNAs (lncRNAs) are a group of non-coding RNAs longer than 200 nucleotides with no protein coding ability. The roles of lncRNAs in various diseases, especially cancer, has attracted extensive attention. LncRNAs, like miRNAs, have different levels of expression in normal tissues and cancer tissues. High expression of some lncRNAs can promote the progression of lung cancer, while high expression of some lncRNAs can inhibit lung cancer angiogenesis and play an anticancer role. Therefore, lncRNAs have attracted more and more attention in recent years.

**Figure 4:**
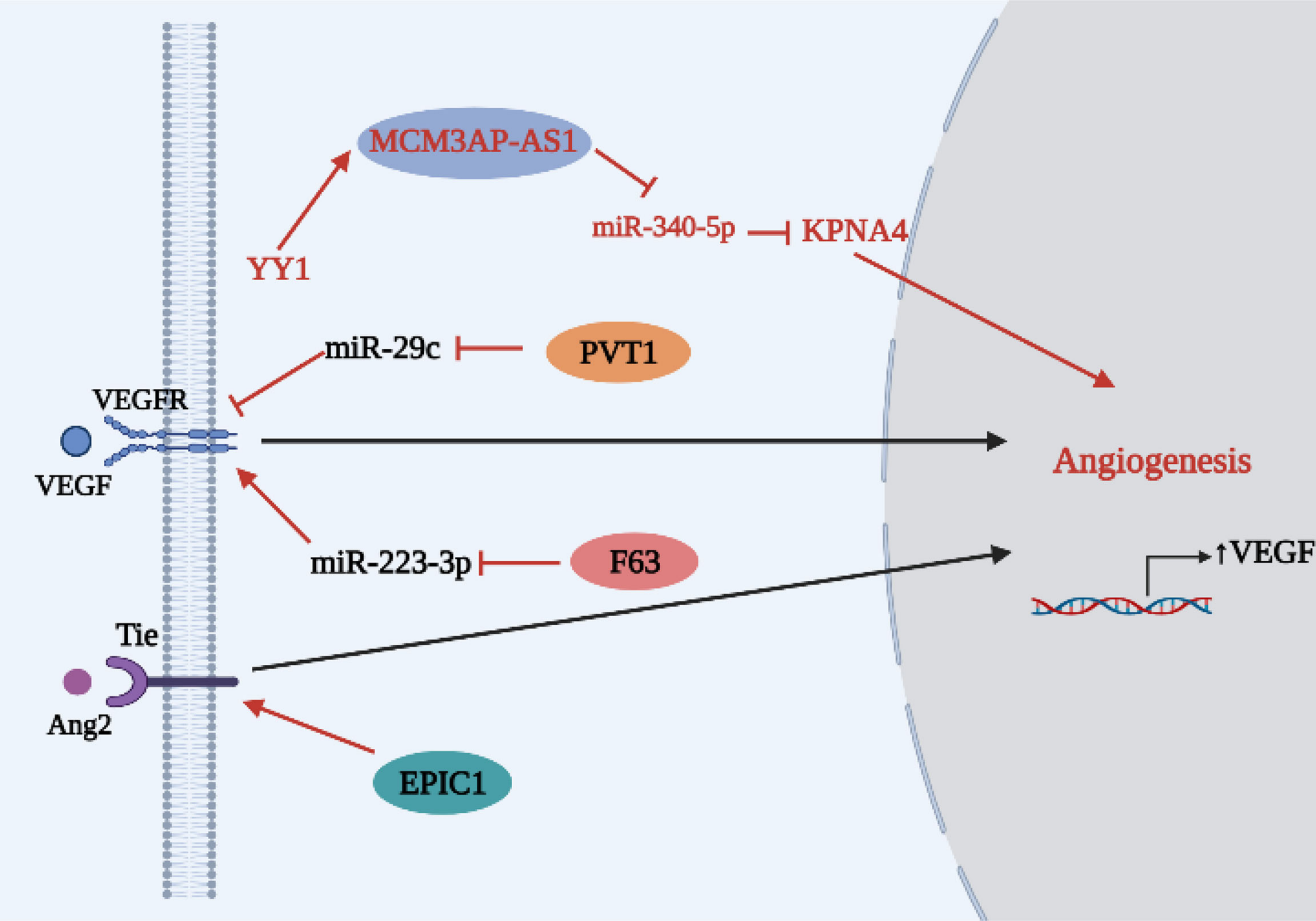
Relationship between lncRNAs and angiogenesis in lung cancer.

As shown in **Table 2** and **Figure 4**, Li et al. (52) studied the lncRNA MCM3AP antisense RNA 1 (MCM3AP‐AS1) and found that silencing of MCM3AP-AS1 decreased the expression of angiogenic related proteins (VEGF, Ang1 and FGF2) in lung cancer cells, weakening angiogenesis ability. Further studies showed that MCM3AP‐AS1 is a downstream target of YY1; YY1 mediates MCM3AP‐AS1 overexpression, and MCM3AP‐AS1 overexpression can inhibit downstream miR-340‐5p expression, accelerating lung cancer angiogenesis and progression through the miR-340-5p/KPNA4 axis. Therefore, MCM3AP‐AS1 overexpression can up-regulate expression of the YY1/MCM3AP‐AS1/miR-340-5p/KPNA4 regulatory axis and promote angiogenesis by increasing secretion of VEGF and other pro-angiogenic factors. PVT1 is a carcinogenic lncRNA that is overexpressed in many cancers (56). Mao et al. (53) found that overexpression of PVT1 in NSCLC can inhibit downstream expression of miR-29c while increasing expression of VEGF and CD31, which are closely related to angiogenesis and poor prognosis of NSCLC. In addition, the lncRNA EPIC1 is significantly upregulated in NSCLC tissues and cell lines, where it stimulates the formation and proliferation of HUVEC tubes and promotes tumor angiogenesis by activating the Ang2/Tie2 pathway (54). Ang2 has been reported to disrupt vascular stability and act synergistically with VEGF-A to promote angiogenesis (57). Similarly, lncRNA LINC00667 stabilizes VEGF-A through EIF4A3 and promotes proliferation, migration and pathological angiogenesis in NSCLC (58). However, Qin et al. (55) found the novel tumor suppressor lncRNA F630028O10Rik (lncRNA F63), which can inhibit VEGFA secretion and EC migration, invasion and duct formation by regulating the F63/miR-223-3p/VEGF axis to inhibit lung cancer development.

**Table 2:** Research progress of lncRNAs in angiogenesis in lung cancer.

↑lncRNAs	Signaling pathway	Angiogenesis indication
MCM3AP-AS1 (52)	YY1/MCM3AP-AS1/miR-340-5p/KPNA4	↑VEGF, Ang1, FGF2
PVT1 (53)	PVT1/miR-29c/VEGF	↑VEGF, CD31
EPIC1 (54)	↑Ang2/Tie2	↑Ang2, VEGF-A
F63 (55)	↓F63/miR-223-3p/VEGF	↓VEGF-A, VEGFR-2

↑: up-regulation. ↓: down-regulation.

LncRNAs, like miRNAs, can act as both tumor suppressors and carcinogens *in vivo* and can regulate angiogenesis through different angiogenesis pathways. Previous studies on lncRNAs remain relatively nascent, and much research remains to be performed.

### Proteins

*In vivo*, enzymes, polypeptides and proteins of various types, structures and roles, participate in the biological activities of the body. Therefore, they are highly valued in various fields. In recent years, proteins have also attracted attention in the field of lung cancer angiogenesis.

**Figure 5:**
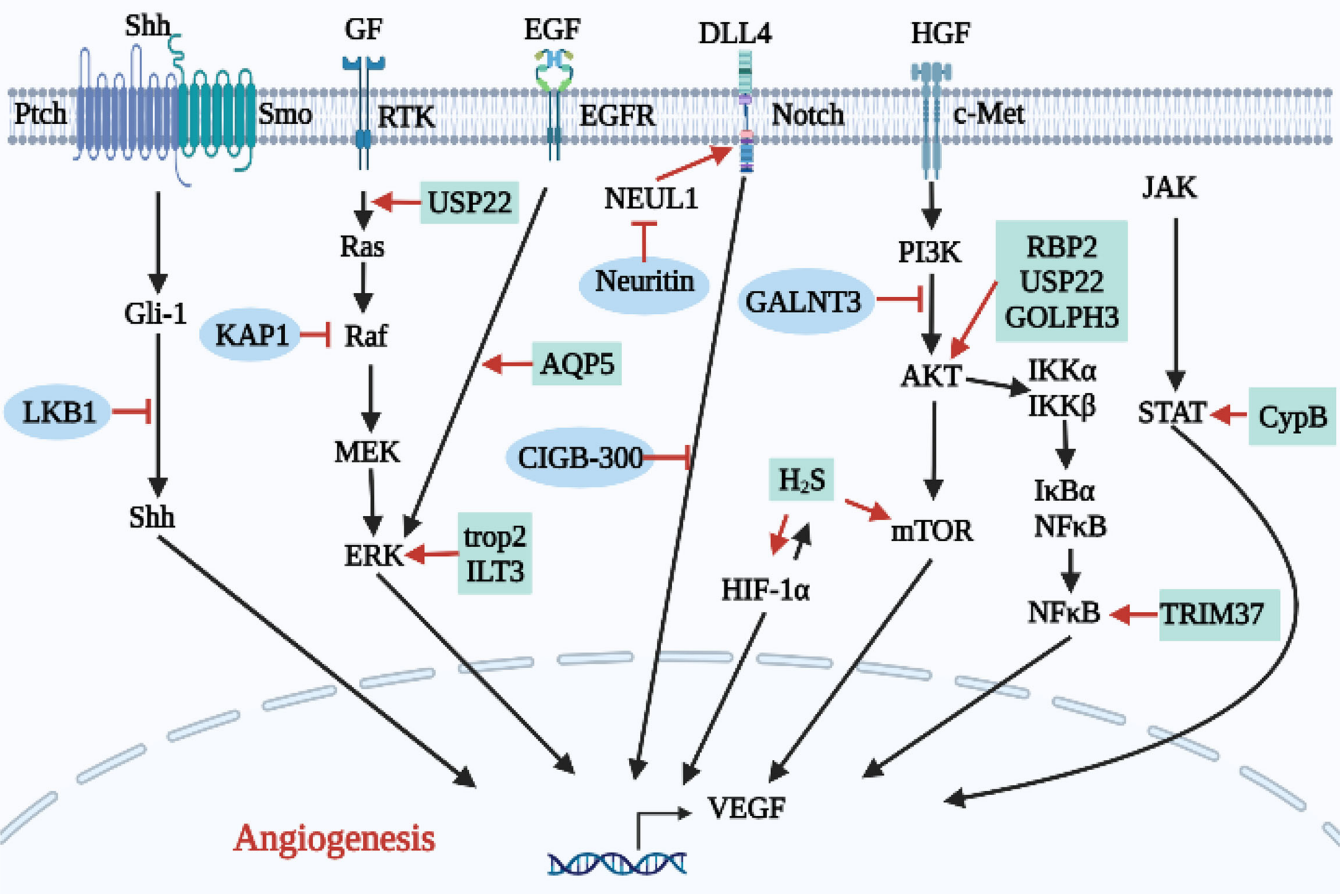
Relationship between proteins and other biomolecules and angiogenesis in lung cancer.

As shown in **Table 3** and **Figure 5**, overexpression of some proteins can inhibit lung cancer angiogenesis by inhibiting angiogenesis signaling pathways.

**Table 3:** Research progress of proteins in angiogenesis in lung cancer.

↑Proteins	Signaling pathway	Angiogenesis indication
LKB1 (59)	↓Shh	↓Shh, Ptch, Smo, Gli ↓HUVECs, VEGF, MMP-2, MMP-9
KAP1 (60)	↓c-Raf/MEK/ERK	↓angiogenesis
Neuritin (61)	↓DLL4/Notch	↑nonfunctional angiogenesis
CIGB-300 (62)	↓Notch/VEGF	↓Notch, VEGF
GALNT3 (63)	↓c-Met/AKT	↓angiogenesis
CypB (64)	↑STAT3	↑VEGF, Ang2
trop2 (65)	↑ERK1/2	↑HUVECs, angiogenesis
ILT3 (66)	↑ApoE-ILT3/SHP2/SHIP1/ERK1/2	↑VEGF-A
USP22 (67)	↑K-Ras, AKT, ERK	↑c-Myc, VEGF
AQP5 (68)	↑EGFR/ERK1/2/VEGF ↑HIF-1α	↑HIF-1α, VEGF
TRIM37 (20)	↑NF-κB	↑VEGF, MMP-9, c-Myc
RBP2 (69)	↑PI3K/AKT ↑HIF-1α/VEGF	↑HIF-1α, VEGF
GOLPH3 (70)	↑AKT ↑Wnt/β-Catenin	↑VEGF

↑: up-regulation. ↓: down-regulation.

The silencing of liver kinase B1 (LKB1) increased levels of VEGF, MMP-2 and MMP-9, enhanced HUVEC activity and migration, and promoted angiogenesis (59). In addition, after LKB1 silencing, mRNA levels of key molecules in the Shh signaling pathway, such as Shh, Ptch, Smo and Gli1, were increased, and nuclear translocation of Gli1 was enhanced, suggesting that LKB1 can inhibit the Shh signaling pathway, thus inhibiting angiogenesis in lung cancer. KAP1 is a universal co-inhibitor of KRAB domain zinc finger proteins (KRAB-ZFPs), which represent the largest group of transcription inhibitors in higher organisms (71). KAP1 is known to bind C-Raf, an endogenous inhibitor of the C-Raf/MEK/ERK signaling pathway and lung cancer angiogenesis is inhibited (60). Neuritin is more highly expressed in para-cancer tissues than in lung cancer tissues. Neuritin interacts with neuralized E3 ubiquitin protein ligase 1 (NEURL1), which is a positive regulator of the Notch1 signaling ligand. Neuritin inhibits the Notch1 signaling pathway by inhibiting NEURL1 andreduces the expression of related angiogenic factors in HUVECs by inhibiting the DLL4/Notch signaling pathway (61). Similarly, CIGB-300 is an anti-casein kinase 2 (CK2) peptide that inhibits the expression of Notch and VEGF in ECs by regulating the Notch/VEGF signaling pathway (62). Polypeptide N-acetyl-galactosaminyltransferases (GALNTs) inhibits angiogenesis and invasion of myeloid-derived suppressor cells (MDSCs) by inhibiting the c-Met/AKT signaling pathway, preventing the generation of optimal TME and inhibiting lung cancer progression (63).

Similarly, as shown in **Table 3** and **Figure 5**, over expression of some proteins promotes angiogenesis in lung cancer.

Previous studies have suggested that the expression of Cyclophilin B (Cyp B) in the serum of pancreatic cancer patients is significantly higher than in that of healthy volunteers (72), but the role of Cyp B in NSCLC remains unclear. Teng et al. (64) showed that Cyp B silencing can significantly inhibit A549 proliferation, reduce expression of the angiogenesis-related proteins VEGF and Ang2, and inhibit the proliferation, migration, invasion and angiogenesis of NSCLC cells by regulating the STAT3 signaling pathway. Tumor-associated calcium signal transducer 2 (trop2) has been confirmed to be associated with tumor proliferation and invasion of NSCLC. Down-regulation of trop2 can significantly inhibit the tube-forming ability of HUVECs. It has been further demonstrated that trop2 promotes NSCLC angiogenesis through the ERK1/2 signaling pathway (65). Similarly, Li et al. (66) found that Immunoglobulin-like transcription 3 (ILT3) is enriched in human NSCLC cells, where it activates the SHP2/SHIP1/ERK1/2 axis through interactions with apolipoprotein E (ApoE), thereby inducing VEGF-A expression and promoting tumor angiogenesis. Ubiquitin specific peptidase 22 (USP22) is a ubiquitin-hydrolase that catalytically removes the monoubiquitin portion of histone H2B (H2Bub1). Frequent loss of H2Bub1 is significantly associated with the invasive tumor biology of lung adenocarcinoma (73, 74). Therefore, USP22 gene knockout significantly inhibits angiogenesis, proliferation, and expression of AKT, K-Ras, ERK and c-Myc in A549 and H1299 cells, while also inhibiting the development of NSCLC (67). Aquaporin 5 (AQP5) which was significantly elevated in H1299 cells, where it may participate in the regulation of lung cancer cell migration and angiogenesis through the EGFR/ERK1/2/VEGF signaling pathway (68). Therefore, down-regulation of trop2, ILT3, USP22 and AQP5 may represent potential targets for the prevention of lung cancer metastasis and angiogenesis. TRIM37 participates in the k63 polyubiquitination of TRAF2, which represents an important step in the activation of NF-κB signaling pathway. Li et al. (20) found that TRIM37 promotes invasion of NSCLC cells by activating the NF-κB pathway. Upon entering the nucleus, NF-κB promotes the expression of c-Myc, MMP-9 and VEGF, thereby promoting lung cancer angiogenesis. Therefore, TRIM37 is a potential target for anti-angiogenesis therapies. Recent studies of lung cancer have demonstrated that retinoblastoma binding protein 2 (RBP2) overexpression in human NSCLC cell lines can also promote HIF-1α/VEGF pathway-induced NSCLC angiogenesis by activating the PI3K/AKT pathway. Increased VEGF expression will further activate the AKT pathway, leading to a vicious cycle of aggravated NSCLC angiogenesis resulting in the further development of lung cancer (69, 75). Similarly, Golgi phosphoprotein 3 (GOLPH3) can also activate the AKT pathway to promote cancer cell proliferation and inhibit apoptosis (76), in addition to increasing VEGF expression and promoting pulmonary adenocarcinoma angiogenesis through the Wnt/β-catenin signaling pathway (70).

There are also small molecules that play roles in regulating angiogenesis. The role of hydrogen sulfide (H_2_S) in the development of different tumors is controversial. Although lungs, as respiratory organs involved in gas exchange, are often exposed to exogenous H_2_S, the role of H_2_S in the progression of NSCLC has not been studied. Studies have shown (77) that H_2_S-producing enzymes are highly expressed in NSCLC tissues and cells, and inhibition of H_2_S-producing enzymes can inhibit the growth of NSCLC cells. We also found that HIF-1α and H_2_S mutually promote angiogenesis through the VEGFR-2/mTOR signaling pathway, and H_2_S promotes angiogenesis through the VEGFR-2/mTOR signaling pathway. Expression of VEGF-A can also be promoted by high expression of HIF-1α, ultimately promoting angiogenesis.

Recently, more and more studies have been performed on carcinogenic/anticancer regulators that target various parts of the angiogenesis signaling pathway in lung cancer. These regulators have some common features. For example, the expression of carcinogenic factors in lung cancer tissues is higher than that in adjacent tissues, while the expression of tumor suppressor factors exhibits the opposite pattern. The difference in the expression of these regulators between cancer and para-cancer tissues is a useful screening factor. These regulators also regulate angiogenesis by affecting multiple signaling pathways. Therefore, if the levels of these regulators *in vivo* can be effectively regulated, regulation of angiogenesis will also be efficient, reducing instances of drug resistance and mutation. However, these regulators also perform other important functions *in vivo*, including neuroregulatory functions as neurotransmitters. Therefore, it is particularly important to effectively regulate these biomolecules without affecting normal bodily function, a major prerequisite for clinical introduction.

## Novel Anti-Angiogenesis Strategies in Lung Cancer Treatment

### Novel Uses for Existing Drugs

As lung cancer itself is complex and refractory, more and more attention has been drawn to identifying novel uses for existing drugs in cancer treatment. Compared with *de novo* synthesis strategies, attempts to explore anti-cancer potential of existing drugs often yield large amounts of data, reducing the time and cost of pharmacokinetic and toxicity studies and improving success rates. Firstly, the research and development strategies regarding new uses for existing drugs are based on drug side effects. The side effects of some drugs under current indications may indicate a potential therapeutic mechanism for another class of diseases, such as Thalidomide. The second approach is the expansion of original indications, which refers to the extension of initial drug indications to other indications on the basis of the original drug target. This approach is less risky and easier to perform, making it common in this field. For example, Afatinib, a dual EGFR and HER2 inhibitor, was approved by the FDA in 2013 for the treatment of EGFR-mutated metastatic NSCLC and in 2017 for the treatment of locally advanced or metastatic squamous histological NSCLC. Thirdly, new indications can be discovered. Many studies have found that existing drugs often act on multiple targets, some of which have antitumor activities. Therefore, maximizing the effects of existing drugs will continue to attract interest in future research.

#### Chemical Medicine

Chemical medicine is the most commonly used and diverse type of medicine for tumor treatment. Their chemical components and indications are relatively clear, so they are particularly suitable for the study of novel applications for existing drugs.

**Figure 6:**
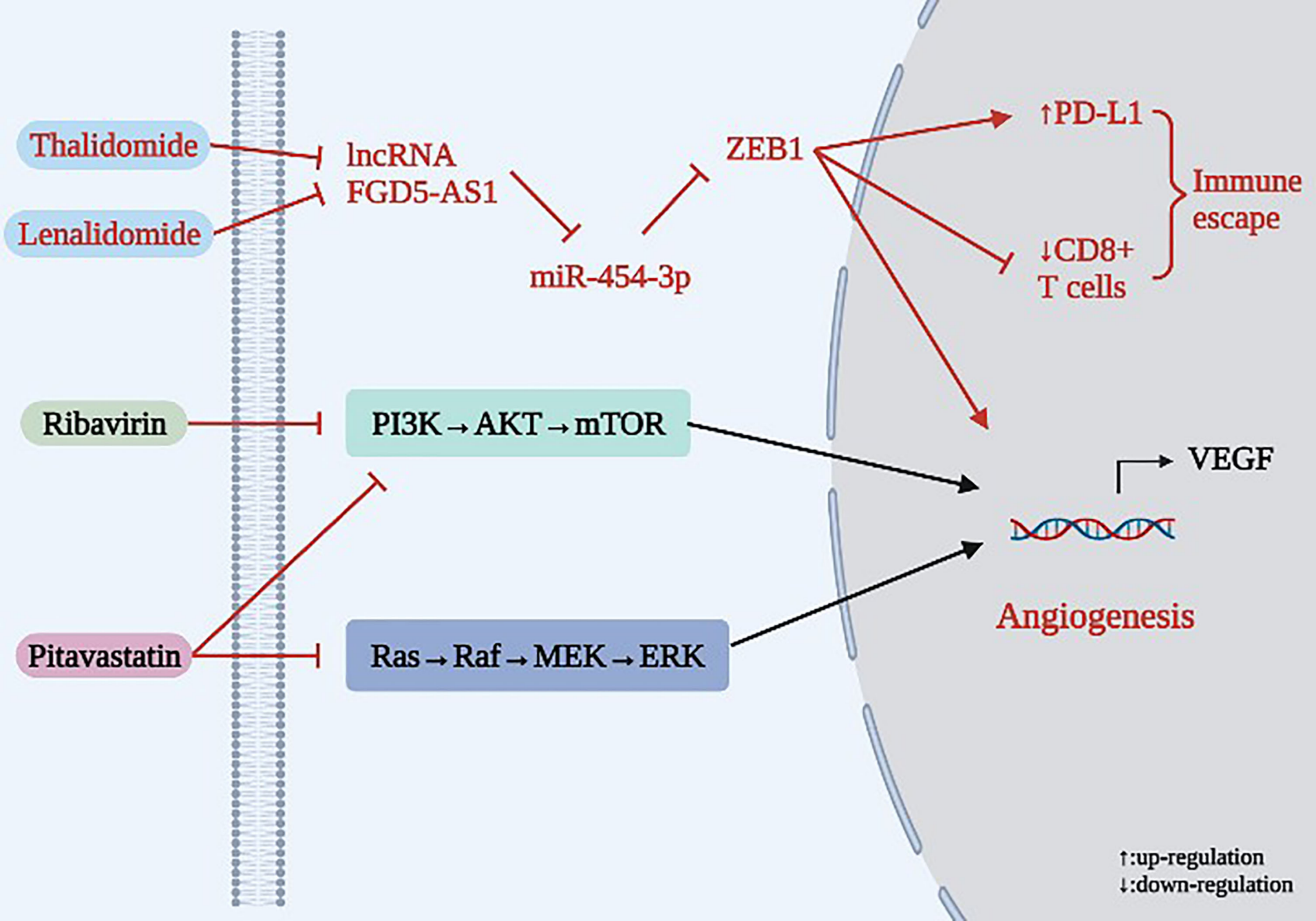
Mechanism of anti-angiogenesis for existing chemical medicine in lung cancer.

As shown in **Table 4** and **Figure 6**, Thalidomide was originally developed to treat epilepsy, then was employed as a sleep aid before becoming widely used as an antiemetic drug for pregnant women. While it was later found to cause infant deformities and was banned in many countries, other effects of thalidomide are still being defined. Xia et al. (78) showed that Thalidomide inhibits angiogenesis and immune escape in NSCLC, exhibiting a dual anticancer role. In terms of mechanism, high expression of lncRNA FGD5-AS1 enhances the proliferation and invasion of NSCLC cells. In patients, FGD5-AS1 expression has been negatively correlated with miR-454-3p expression. The expression of ZEB1 in tumor tissues of NSCLC patients was positively correlated with FGD5-AS1 and negatively correlated with miR-454-3p. Later, this study found that the FGD5-AS1/miR-454-3p/ZEB1 axis can activate expression of PD-L1 in NSCLC cells, leading to the inactivation of CD8+ T cells and immune escape of NSCLC. However, expression of VEGFA and PD-L1, which are key regulatory factors promoting tumor angiogenesis and immune escape, can be significantly inhibited by silencing FGD5-AS1. Therefore, FGD5-AS1 is an oncogene, while miR-454-3p is a tumor suppressor gene. In this study, Thalidomide inhibited expression of VEGFA and PD-L1 by inhibiting the lncRNA FGD5-AS1/miR-454-3p/ZEB1 axis, thereby inhibiting angiogenesis and immune escape in NSCLC. Lenalidomide is an important derivative of Thalidomide that has been demonstrated to have anti-tumor, immunomodulatory and anti-angiogenic effects, among others. Ribavirin is a common antiviral drug in clinical practice and represents an attractive candidate for the treatment of lung cancer. Due to its ability to target eukaryotic translation initiation factor 4E (eIF4E), which plays a critical role in the translation of VEGF and other pro-angiogenic genes, Ribavirin has exhibited strong anticancer activity against various cancers. Studies have found that Ribavirin is active against lung cancer cell lines regardless of molecular and cellular heterogeneity, and it acts on lung cancer cells by inhibiting eIF4E and mTOR signaling pathways, thereby inhibiting eIF4E-mediated protein translation and exerting an anti-angiogenic effect (79). The cholesterol-lowering drug Pitavastatin can inhibit lung cancer-related EC migration and morphologic changes without affecting adhesion, thus demonstrating a role in inhibiting lung cancer cell growth and angiogenesis. Mechanically, Pitavastatin inhibits angiogenesis in lung cancer both *in vitro* and *in vivo* by inhibiting the allyl-dependent Ras/Raf/MEK and PI3K/AKT/mTOR signaling pathways. This study was the first to demonstrate the inhibitory effect of Pitavastatin on the Ras-mediated signaling pathway (80).

**Table 4:** Research progress of existing chemical medicine in angiogenesis in lung cancer.

Chemical medicine	↓Signaling pathway	↓Angiogenesis indication
Thalidomide (78)	lncRNA FGD5-AS1/miR-454-3p/ZEB1	VEGF-A, PD-L1
Ribavirin (79)	eIF4E/p70S6K mTOR	VEGF
Pitavastatin (80)	Ras/Raf/MEK PI3K/AKT/mTOR	angiogenesis

↓: down-regulation.

#### Natural Compounds

Natural compounds exhibit high efficiency, low toxicity and minimal side effects in tumor treatment, so they have great potential in the research field of drug repurposing.

**Figure 7:**
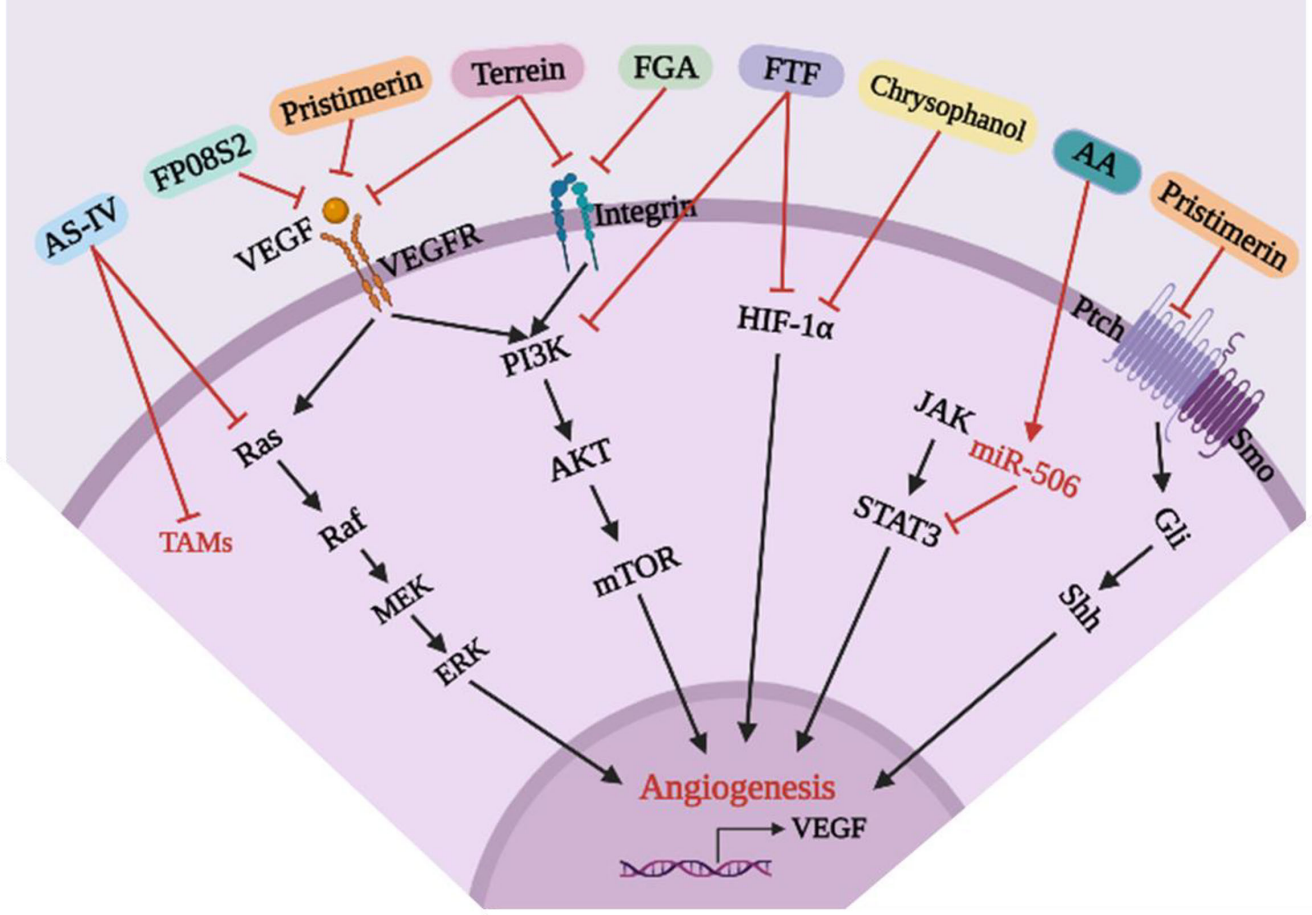
Mechanism of anti-angiogenesis for existing natural compounds in lung cancer.

As shown in **Table 5** and **Figure 7**, Pristimerin is an active natural compound isolated from *Astragalus membranaceus* with anti-inflammatory, anti-peroxidation and anti-microbial effects (89). Subsequent studies showed that Pristimerin inhibited the proliferation, migration and invasion of HUVECs, inhibited the formation of tubular structures, inhibited cell adhesion and recruitment, reduced expression of CD31, and inhibited Shh-induced angiogenesis *in vivo* and *in vitro*. Mechanically, Pristimerin may first inhibit Shh/Gli1 activation, then inhibit downstream VEGF/VEGFR2, AKT, and ERK signaling pathways, thereby inhibiting angiogenesis in lung cancer. However, the mechanism by which Shh/Gli1 regulates the VEGF/VEGFR2 signaling pathway needs to be further studied (81). Similarly, sulfated fucoidan FP08S2 inhibits lung cancer cell growth *in vivo* by targeting VEGFR2/VEGF, blocking the VEGFR2/ERK/VEGF signaling pathway and reducing VEGF expression to block angiogenesis (82). Terrein is a natural substance produced by microorganisms with a variety of potential attributes, including anti-inflammatory and anticancer effects. Terrein can simultaneously inhibit VEGF-A/VEGFR-2 and Integrin/focal adhesion kinase (Integrin/FAK) in A549 cells, in addition to reducing protein levels of downstream mediators, such as PI3K, AKT, mTORC1, and P70S6K, and expression of MMP-2 and MMP-9. Mechanically, Terrein inhibits the PI3K/AKT/mTOR signaling pathway by inhibiting HIF-1α and VEGF translation and angiogenesis in lung cancer (83). Similarly, studies have shown that fibrinogen α (FGA) can inhibit the proliferation, migration and angiogenesis of lung cancer cells by inhibiting the Integrin/AKT/mTOR signaling pathway, promoting cell apoptosis and exerting an anticancer role (84). Fritillariae Thunbergii Flos (FTF) is used to treat bronchitis. In recent years, it has been used as a lung cancer treatment. Studies have shown that the anti-tumor effects of FTF may be mediated by HIF-1α and the PI3K/AKT signaling pathway, inhibiting angiogenesis in lung cancer and thereby inhibiting tumor development (85). Chrysophanol, an anthraquinone, is a natural active ingredient that exhibits antibacterial activity against a variety of bacteria and can relieve cough. Recent studies have shown that Chrysophanol can inhibit the expression of VEGF, MMP-2, MMP-9, CD31 and HIF-1α by inhibiting the HIF-1α/VEGF signaling pathway and can inhibit the formation and proliferation of HUVEC tubes, angiogenesis, and tumor growth (86). TME plays an important role in the pathogenesis of lung cancer. Tumor-associated macrophages (TAMs) promote growth, invasion, metastasis and angiogenesis of cancer cells and are one of the major tumor-infiltrating immune cells with immunosuppressive activity (90). Therefore, TAMs have been considered as potential targets for adjuvant anti-tumor therapy. Astragaloside IV (AS-IV), a natural saponin extracted from *Astragali* radix, has been reported to have anti-inflammatory, anti-cancer, anti-oxidative, and immune-regulatory effects. Xu et al. (87) investigated the relationship between macrophage polarization and the *in vitro* and *in vivo* antitumor effects of AS-IV and found that AS-IV directly acted on macrophages, inhibited macrophage polarization to the M2 phenotype, and inhibited invasion, migration and angiogenesis of lung cancer cells by regulating the AMPK signaling pathway. Alginic acid (AA) is a naturally occurring polysaccharide aldehyde acid. Recent studies have demonstrated its antiallergic and anti-inflammatory properties. Studies have found that AA has a significant inhibitory effect on NSCLC-induced angiogenesis in tube formation and xenotransplantation models (88). Subsequent studies have shown that AA inhibits NSCLC-induced angiogenesis by activating miR-506 expression and inhibiting expression of VEGF-A, a key factor in the STAT3 signaling pathway and angiogenesis.

**Table 5:** Research progress of existing natural compounds in angiogenesis in lung cancer.

Natural compounds	↓Signaling pathway	↓Angiogenesis indication
Pristimerin (81)	Shh/Gli VEGF/VEGFR-2	Shh, Gli, ERK, AKT VEGF, CD31
FP08S2 (82)	VEGFR2/ERK/VEGF	HIF-1α, VEGF
Terrein (83)	PI3K/AKT/mTOR MEK/ERK	PI3K, AKT, mTORC1, p70S6K HIF-1α, VEGF, MMP-2, MMP-9
FGA (84)	Integrin/AKT/mTOR	angiogenesis, cell proliferation and migration
FTF (85)	HIF-1α PI3K/AKT	angiogenesis
Chrysophanol (86)	HIF-1α/VEGF	VEGF, CD31, HIF-1α, HUVECs, MMP-2, MMP-9
Astragaloside IV (87)	AMPK	tumor progress and metastasis
Alginic acid (88)	miR-506/STAT3/VEGF-A	STAT3, VEGF-A

↓: down-regulation.

In conclusion, some common chemical medicine and natural compounds demonstrate the potential to regulate lung cancer angiogenesis. If we can study and summarize the potential of these chemical medicine and natural compounds for clinical use, patients can obtain effective treatment at a reduced cost, while also limiting the serious toxicity and side effects of anti-cancer drugs. For those who develop new drugs, there is a wider research scope, as existing molecular structures can be leveraged to develop more effective drugs, leading to greater benefits with less investment and less risk.

### New Drug Delivery Systems

An ideal development would be the identification of dual-domain therapeutic agents that can simultaneously kill tumors and inhibit their blood supply microenvironment, combining the effects of different drugs in the previous combination regimen to achieve an “A+” effect. VEGF small interfering RNA (siRNA) can precisely and effectively silence VEGF expression, block the VEGF signaling pathway, lead to a significant reduction in tumor blood vessels, and inhibit tumor growth and metastasis. Etoposide (ETO) is a poorly soluble topoisomerase II inhibitor that can induce tumor cell apoptosis and has been reported as an effective chemotherapy drug for NSCLC; however, the non-selective accumulation of ETO *in vivo* has serious side effects on normal tissues (91). Therefore, it is urgent to develop a safe and efficient drug delivery system. Li et al. (92) constructed a multifunctional co-loading liposome (PHCL-lip) that simultaneously loaded siVEGF and ETO. *In vivo* pH trigger and EPR effects promoted deep-tumor infiltration of the drug-loading liposome, and drug release at the tumor site resulted in ETO-induced tumor cell apoptosis and siVEGF-induced VEGF signaling pathway blockade. Compared with monotherapy, combined therapy with ETO and siVEGF exhibits a good long-term inhibitory effect on tumor proliferation and angiogenesis of *in situ* NSCLC with low biotoxicity. Therefore, co-administration of siVEGF and ETO using the system developed in this study can simultaneously inhibit tumor growth and block tumor neovascularization. This study used a combination of anti-angiogenesis therapy and chemotherapy to treat NSCLC, which has important clinical transformation potential.

It is worth noting that anti-angiogenesis therapy is traditionally a very reasonable treatment strategy for lung cancer. However, tumor blood vessels are abnormal and often exhibit structural and functional impairments and instability. Therefore, the TME is not only hypoxic and acidic, but is also surrounded by high interstitial pressure, meaning that drugs are not easily accessible, and efficacy is low (93). Traditional antiangiogenic approaches often lead to extreme hypoxia in tumors, ultimately leading to increased drug resistance, local invasion, and more distant metastasis (94). Therefore, at present, the clinical efficacy of anti-angiogenesis drugs is limited, and some inhibitors are even completely ineffective or toxic. New, safer and more effective drugs are urgently needed. Qin et al. (95) recently showed that Resveratrol (RSVL) combined with Gemcitabine (GEM) has stronger tumor growth inhibition than GEM alone. In terms of mechanism, this study suggests that RSVL promotes tumor microvascular growth by down-regulating expression of endothelin and activating the ERK signaling pathway, leading to increased blood perfusion and increased drug entry into the tumor, ultimately enhancing the anticancer effect of GEM. The results of this study suggest that RSVL has potential clinical application in improving the efficacy of anticancer drugs against lung cancer, and the ingestion of RSVL may be beneficial during chemotherapy for the treatment of lung cancer.

### Antiangiogenic Therapy Combined With Other Anti-Cancer Therapies

Although multiple lines of anti-angiogenesis drugs are now available, monotherapy with angiogenesis inhibitions often fails to achieve desired results and resistance eventually occurs in almost all lung cancer patients. Therefore, two or more anti-angiogenesis drugs targeting different angiogenesis signaling pathways are often combined to achieve better anticancer efficacy (defining as “A + A” strategy) (96). For example, the combination therapy of bevacizumab and erlotinib was recently approved for treatment of NSCLC with EGFR-activating mutations and the results were further confirmed by a phase 3 study (NEJ026) (97). Traditional angiogenesis-targeting therapy is aimed at inhibiting TME, and then delaying tumor growth but not killing the tumor itself. Currently, anti-angiogenesis drugs are often combined with chemotherapy clinically to maintain tumor stability, which is the recognized “A + C” strategy. One study assessed the effect of combining PTX with Apatinib or Bevacizumab, demonstrating that the inclusion of PTX considerably enhanced the antitumor effect of both drugs and prolonged survival compared with either monotherapy (98). However, due to the rapid proliferation of lung cancer cells, the rapid relapse of drug resistance after chemotherapy, and the obvious toxicity of chemotherapy drugs, the application of “A + C” strategy in lung cancer treatment is difficult. Nevertheless, we are still looking for highly effective therapy strategies with low toxicity that can suppress tumor growth powerfully over the long term. An emerging therapy, the “A + I” strategy of combining anti-angiogenesis drugs with immunotherapy, is expected to prolong the survival of lung cancer patients. This is a phase III multicenter randomized controlled clinical trial about the treatment of bevacizumab in combination with atezolizumab for advanced non-squamous NSCLC. The results showed that the atezolizumab plus bevacizumab and paclitaxel/carboplatin (ABCP) treatment achieved greater PFS and OS benefit than bevacizumab plus paclitaxel/carboplatin (BCP) treatment. Based on this study, FDA approved ABCP as first-line treatment of metastatic non-squamous NSCLC patients on December 6, 2018 (99). Besides, Chen et al. reported that in a lung cancer mouse model, the triple combination of anti-VEGF, anti-PD-L1, and radiotherapy therapies more obviously enhances the existing anticancer efficacy (100). In addition, a more ideal strategy is to develop “double-field anti-cancer drugs”, which can inhibit tumor growth and blood supply microenvironment at the same time, achieving the goal of “A +” strategy. Clinically, Anlotinib that is a double-field (inhibiting tumor growth and TME at the same time) and multiple-target (covering the tumor growth and angiogenesis of signaling pathways at the same time) anti-cancer drug, can reduce the incidence of drug resistance. In conclusion, better combination regimens (101) and effective dual-field, multi-target drugs need to be explored to overcome monotherapy-induced resistance.

## Conclusion and Outlook

Angiogenesis plays a key role in the progression of lung cancer. Compared with normal cells, tumor cells have a higher demand for nutrients and require more energy for cell division, metastasis and invasion, so the presence of tumor induces increased angiogenesis. Inhibition of angiogenesis inhibits tumor growth, so anti-angiogenesis therapies have been widely studied in lung cancer in recent years. Clinically, the effects of anti-angiogenesis drugs are not satisfactory at present, and significant drug resistance problems are evident. This may be because there are many pathways involved in tumor angiogenesis, such that inhibition of any one or several may be compensated by other pathways. Moreover, the advantages and disadvantages of inhibiting a certain part of the signal pathway require further study. Therefore, antiangiogenic therapy often combines with other other lung cancer therapeutic approaches to acquire better clinical efficacy. Current basic research regarding angiogenesis inhibitors primarily focuses on discovering new components of the angiogenesis regulator axis and related angiogenesis signaling pathways and screening carcinogenic/anticancer genes, miRNAs, lncRNAs and proteins that regulate pathways and expression of specific targets, with the ultimate goal of inhibiting activation of angiogenesis signaling pathways. These studies seek to explain the mechanism of angiogenesis more clearly and inhibit angiogenesis at its root. Research into repurposing existing drugs fully leverages the potential of existing chemical medicine and natural compounds against angiogenesis and provides new ideas for the development of anti-angiogenesis drugs. In addition, due to developments in nano-drug delivery systems, some studies have realized the co-delivery of anti-angiogenesis drugs and chemotherapy drugs. The EPR effect of the tumor and targeting properties of the designed nanoparticles were utilized to accurately deliver chemotherapy drugs and inhibit tumor angiogenesis simultaneously. Increased attention has been paid to targeting angiogenesis in lung cancer treatment. In the future, we believe that anti-angiogenesis therapy will become a key method for lung cancer treatment.

## Author Contributions

YL was responsible for literature search, article writing and manuscript revision. ML, SW, and BC were responsible for the revision of article content. CL was responsible for the design of writing ideas and article revision. GL was responsible for giving guidance to the topic selection and article content. All authors contributed to the article and approved the submitted version.

## Funding

This study was supported by Beijing Hope Run Special Fund of Cancer Foundation of China (LC2020L03, China) and Beijing Municipal Science and Technology Commission (Z181100001618003, China).

## Conflict of Interest

The authors declare that the research was conducted in the absence of any commercial or financial relationships that could be construed as a potential conflict of interest.

## Publisher’s Note

All claims expressed in this article are solely those of the authors and do not necessarily represent those of their affiliated organizations, or those of the publisher, the editors and the reviewers. Any product that may be evaluated in this article, or claim that may be made by its manufacturer, is not guaranteed or endorsed by the publisher.
